# Arteriovenous Fistula Between the Hepatic Artery and the Hepatic Vein

**DOI:** 10.1155/1989/36396

**Published:** 1989

**Authors:** John M. Howard, M. Malafa, Robert J. Coombs, Anthony M. Iannone

**Affiliations:** 1 Departments of Surgery , Radiology and Neurology Medical College of Ohio C.S. #10008 Toledo Ohio 43699 USA; 2 Department of Surgery Medical College of Ohio C.S. 10008 Toledo OH 43699 USA

## Abstract

A patient is presented with multiple vascular anomalies in the branches of the celiac axis as well as in the portal vein and its branches. Apparently, unique in the literature is the presence of a large arteriovenous fistula between the hepatic artery and one of the hepatic veins. The anomalies are presumed to be congenital in origin.


*HPB Surgery* 1989, Vol. 1, pp. 155–160
Reprints available directly from the publisher
Photocopying permitted by license only

© 1989 Harwood Academic Publishers GmbH
Printed in Great Britain

CASE REPORT

### ARTERIOVENOUS FISTULA BETWEEN THE HEPATIC ARTERY AND THE HEPATIC VEIN

JOHN M. HOWARD,*, M. MALAFA, ROBERT J. COOMBS, and ANTHONY M. IANNONE

*Departments of Surgery, Radiology and Neurology Medical College of Ohio C.S.* #10008, Toledo, Ohio 43699, USA

*(Received 12 February 1988; in final form 24 May 1988)*

A patient is presented with multiple vascular anomalies in the branches of the celiac axis as well as in the portal vein and its branches. Apparently, unique in the literature is the presence of a large arteriovenous fistula between the hepatic artery and one of the hepatic veins. The anomalies are presumed to be congenital in origin.

KEY WORDS: Arteriovenous fistula, hepatic artery, hepatic vein

This report describes a fistula, apparently congenital, between the hepatic artery and an hepatic vein. As the authors could find no report of a similar anomaly, the following case review is presented.

An 80 year old white female was admitted to the Medical College of Ohio Hospital in 1987 because of generalized seizures. The seizures consisted of catatonic, jerking movements of her upper and lower extremities, lasting 3–4 hours. Several months prior to this admission, dysthymic rhythmic movements of her lips and tongue had been noted. She had then been hospitalized in another hospital because of the seizures and for the treatment of pulmonary emphysema. She was found to be hypertensive and hypothyroid. A tentative diagnosis of idiopathic epilepsy has been made and she had been placed on anticonvulsant therapy.

Except for the above findings, her physical examination was negative. Neither her liver nor spleen was palpable. No vascular bruit could be heard over the abdomen. She was not jaundiced nor was there ascites.

A careful review of her history gave no evidence of significant trauma, jaundice, hepatic disease, nor acute illness of any type. Her family history, although incomplete, was negative for hepatic, vascular or other anomalies.

Her biochemical and hematological profiles were essentially normal except that while septic, her SGOT, SGPT, LDH and alkaline phosphatase levels were mildly elevated. Her serum bilirubin level was normal.

Following a normal electroencephalography and CT scan of the brain, a neurological diagnosis of tardine dyskinesia was made. Meanwhile, she became febrile and consideration was given to the possibility of an abdominal infection.

*Please direct all correspondence to: John M. Howard, M.D., Department of Surgery, Medical College of Ohio, C.S. 10008, Toledo, OH 43699 (419) 381–3586

### RADIOGRAPHIC FINDINGS

An ultrasound scan of the gallbladder revealed a sonolucent lesion within the right lobe of the liver (Figure 1) which, in light of the clinical findings, was initially interpreted as a possible hepatic abscess. A CT scan of the abdomen (Figures 2 A & B), showed a 9.0 × 7.5 cm low attenuation lesion within the anterior aspect of the right lobe of the liver. Following intravenous contrast a rounded, enhancing structure was noted within its central portion. A contrast outlined a linear shaped structure which coursed inferiolaterally to the spherical structure. This was interpreted as a vascular malformation with a surrounding area of necrosis, requiring arteriography for better delineation.

The celiac axis angiogram (Figure 3A) revealed an anomalous right hepatic artery originating as a separate trunk proximal to the origins of the main hepatic and splenic arteries. In the arterial phase, numerous fusiform and occasional small saccular aneurysms were noted within the gastroepiploic, splenic and hepatic arteries. Fed by the right hepatic artery, the contrast outlined the above spherical mass which was visualized early in the arterial phase and corresponded to the findings described on both the CT and ultrasound examinations. The linear enhancing structure on the CT was found to be a large hepatic vein which drained promptly into the inferior vena cava during the arterial phase of this study. A second large malformation was noted inferior-medially to the above described lesion. This lesion reached its peak contrast enhancement during the venous phase

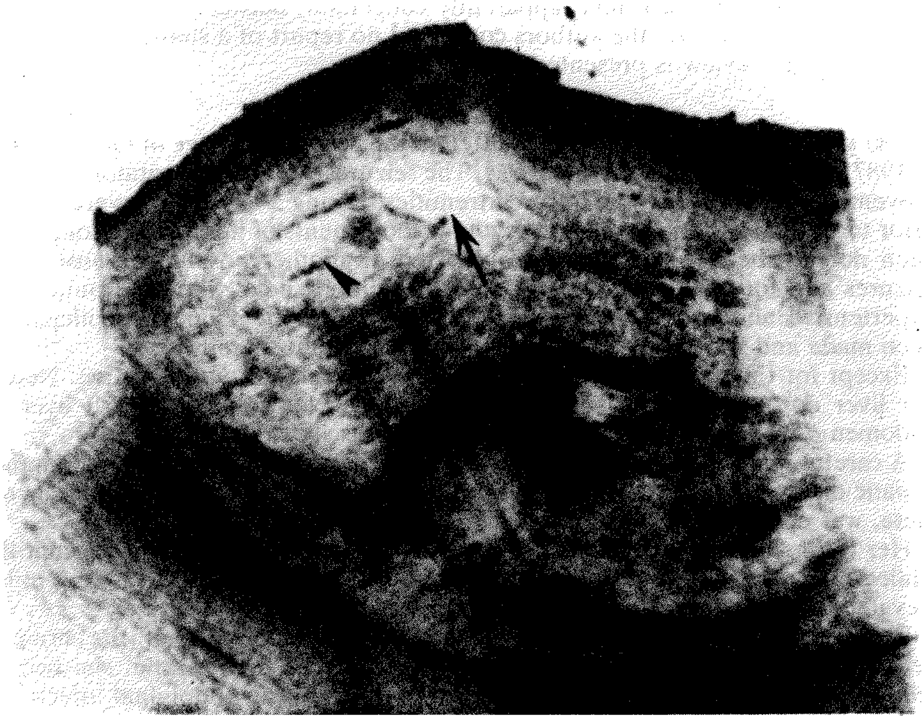

**Figure 1** Transverse sonogram through liver showing hypoechoic lesion in the right lobe with spherical (arrow) and linear (arrowhead) components.

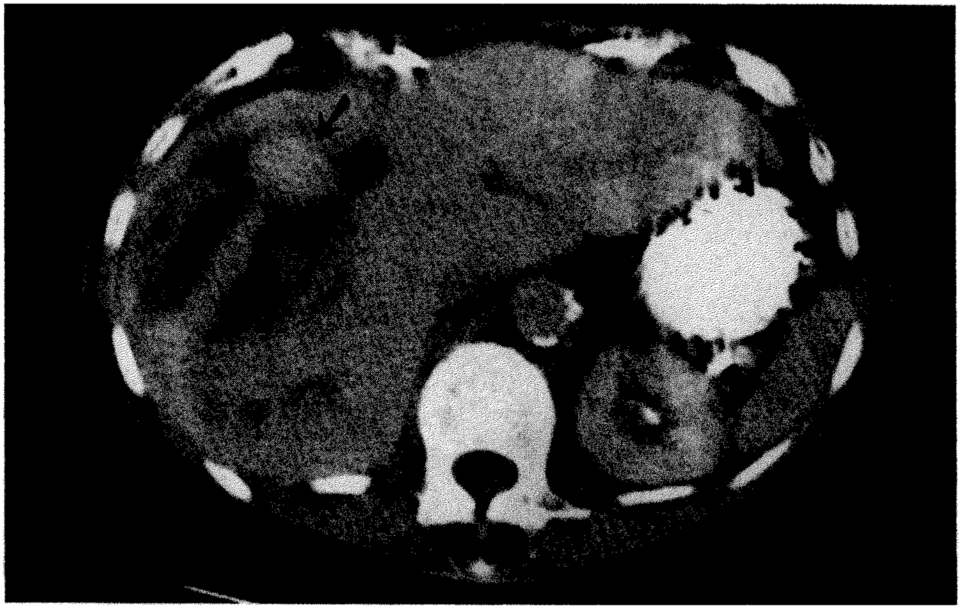

**Figure 2A** CT scan showing enhancing arterial to hepatic venous malformation (arrow) with large draining vein.

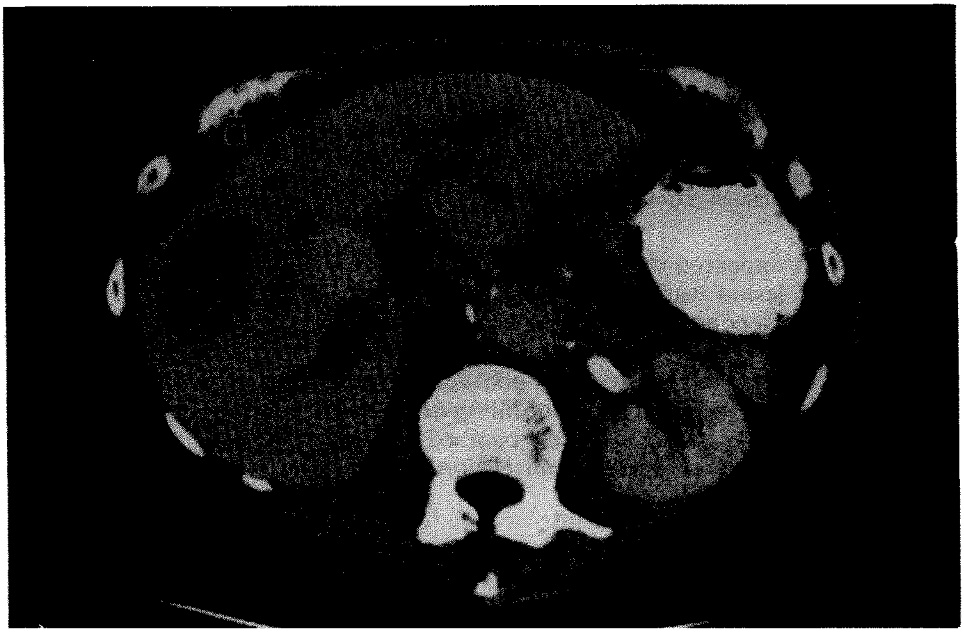

**Figure 2B** Showing additional enhancing arterial venous malformations (arrows).

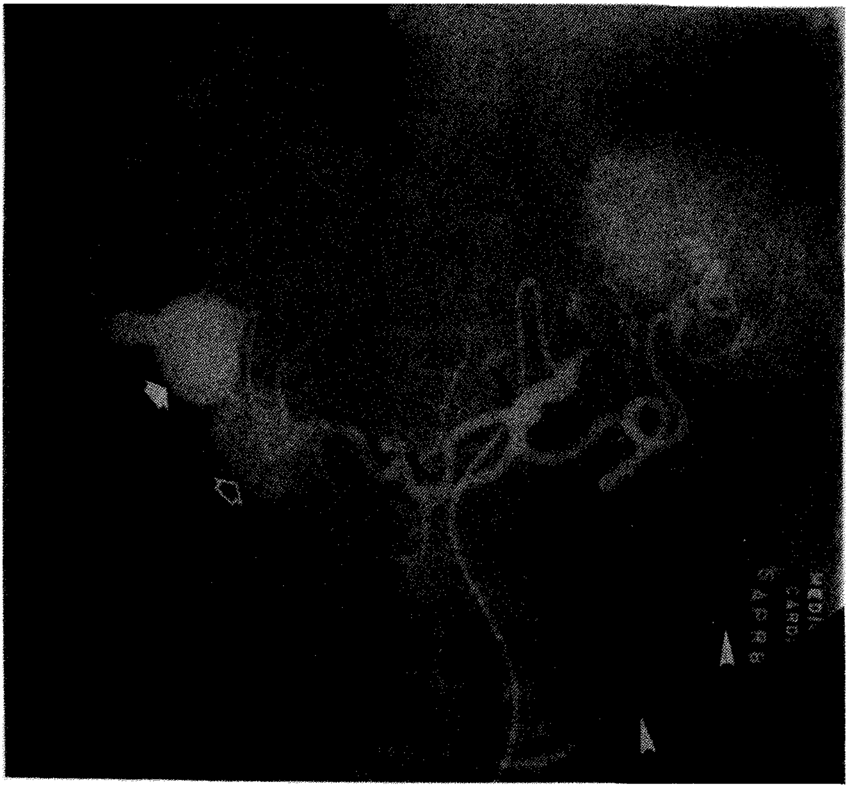

**Figure 3A** Arterial phase showing dense filling of hepatic artery to hepatic venous malformation (arrow) with large draining vein which reaches the inferior vena cava. Partial filling of second malformation (open arrow) is also noted. Multiple small fusiform and saccular aneurysms are seen throughout the arterial distribution (arrowheads).

(Figure 3B). It appeared to be supplied both by the hepatic artery and the portal vein. The first lesion was apparently supplied by an hepatic artery only since it washed out almost completely on the venous phase. Two other spherical malformations were noted on the venous phase. These lesions, in retrospect, were also present on the patient's CT scan. In addition to the malformations noted on the venous phase, note was made of poor filling of the normal intrahepatic portal-vein branches. Tortuous collateral vessels coursed inferiorly from the splenic vessels, suggesting an element of portal hypertension.

### SUBSEQUENT COURSE

The patient was placed on broad spectrum antibiotics, following the culture of E. coli from the blood stream. The source of infection was thought to be the upper urinary tract. She continued to improve and was discharged without evidence of infection and with her seizures under control.

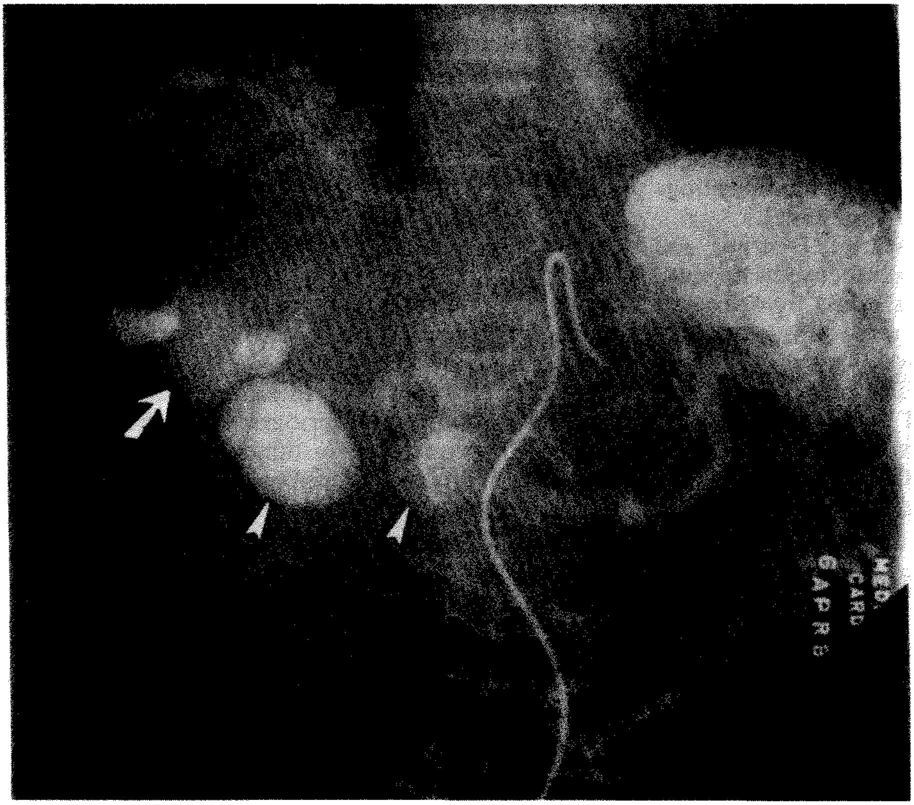

**Figure 3B** Showing venous phase of the angiogram. Washout of the malformation supplied by the artery only is noted (arrow). Additional malformations supplied by the portal vein (arrowheads) are also noted.

She has been followed as an outpatient for the ensuing year and has remained stable without loss of weight, sepsis or new manifestations of any underlying problem. Critical examination of her vascular system has remained otherwise unrevealing. A recent nuclear magnetic resonance study of her brain revealed only evidence of mild atrophy.

### DISCUSSION

Following the CT scan, a diagnostic aspiration of the hepatic lesion had been considered. As subsequently indicated by the arteriogram, intraperitoneal hemorrhage might have ensued. The arteriovenous fistula between the hepatic artery and the hepatic vein was an unsuspected finding prior to the CT scan and arteriogram. Its etiology remains uncertain but the diffuse nature of the malformation and its association with other vascular abnormalities suggest a congenital origin.

Review of the literature reveals numerous reports of fistulae between the hepatic artery and the portal vein, some being the result of congenital anomalies, others being the result of trauma[1,2,3,4,5] including complication of percutaneous transhepatic cholangiography. Portal hypertension sometimes resulted. Multiple aneurysms of the viscera, especially of the renal arteries, are recognized as a manifestation of polyarteritis nodosa[6,7,8,9,10]. Our patient's abdominal aortogram revealed evidence of fibromuscular hyperplasia of the renal arteries but no evidence of a renal artery aneurysm.

Osler-Rendu-Weber syndrome was also considered as it may include arteriovenous communications plus psychiatric disturbances but supporting evidence was not apparent.

Martin, Benzing, and Kaplan[11] reported that a 3 year old was found to have a fistula between a branch of the right hepatic artery and the ductus venosus. This was cured by operative interruption of the arterial branch near the fistula. The anomaly was anatomically different from that presented by our patient but the physiological effects would be qualitatively similar.

The neurologic disorder appeared to the authors to be unusual. Although the CT and NMR scans of the brain were normal, arteriography of the brain would have been of interest. Clinically, it does not seem justifiable.

Consideration was given to the possibility of bacterial vegetations associated with the A–V fistula but this remains conjectural.

### *References*

1. Takayasu, K., Moriyama, N., Shima, T., *et al.* Spontaneous portalhepatic venous shunt via an intrahepatic portal vein aneurysm. *Gastroenterology* **86:** 945–948, 1984.
2. Waes, L.V., Demeulenaere, L., Van Damme, W.V., *et al.* Hepaticoportal fistula and portal hypertension. *Digestive Disease and Sciences* **24:** 565–569, 1979.
3. Foley, W.J., Turcotte, J.G., Hoskins, P.A., *et al.* Intrahepatic arteriovenous fistulas between the hepatic artery and portal vein. *Annals of Surgery* **174:** 849–855,1971.
4. Van Way, C.W. III, Crane, J.M., Riddell, D.H., and Foster, J.H. Arteriovenous fistula in the portal circulation. *Surgery* **70:** 876–890, 1971.
5. Missavage, A.E., Jones, A.M., Walt, A.J., *et al.* Traumatic hepatic arteriovenous fistula causing portal hypertension and variceal bleeding. Journal of Trauma **24:** 355–358, 1984.
6. Herschman, A., Blum, R., and Lee, Y.C. Angiography findings in polyarteritis nodosa. Report of a case. *Radiology* **94:** 147–148, 1970.
7. Oyen, G.W.R., Waer, M., Baert, A.L., *et al.* Case Report. CT demonstration of aneurysms and polyarteritis nodosa. *Journal of Computer Assisted Tomography* **10:** 513–515, 1986.
8. Bron, K.M., Strott, C.A., and Shapiro, A.P. The diagnostic value of angiographic observations in polyarteritis nodosa. *Archives of Internal Medicine* **116:** 450–454, 1965.
9. Pais, S.O. Steiner, R., and Rosenbaum, J.L., *et al.* Multiple aneurysms of the abdominal aorta. *JAMA* **206:** 2737–2739, 1968.
10. Chudacek, Z. Angiographic diagnosis of polyarteritis nodosa of the liver, kidney and mesentery. *British Journal of Radiology* **40:** 864–865, 1967
11. Martin, L.W., Benzing, G., and Kaplan, S. Congenital Intrahepatic Arteriovenous Fistula: Report of a Successfully Treated Case. *Annals of Surgery* **161:** 209–212, 1965.

*Accepted by S. Bengmark 20 May 1988.*

